# Removal of Lead by Merlinoite Prepared from Sugarcane Bagasse Ash and Kaolin: Synthesis, Isotherm, Kinetic, and Thermodynamic Studies

**DOI:** 10.3390/molecules26247550

**Published:** 2021-12-13

**Authors:** Tussaneetorn Chuenpratoom, Khuanjit Hemavibool, Kritsana Rermthong, Suwat Nanan

**Affiliations:** 1 Department of Chemistry, Faculty of Science, Naresuan University, Phitsanulok 65000, Thailand; tussaneetornc@nu.ac.th (T.C.); khuanjitb@nu.ac.th (K.H.); rermthongk@gmail.com (K.R.); 2 Materials Chemistry Research Center, Center of Excellence for Innovation in Chemistry (PERCH-CIC), Department of Chemistry, Faculty of Science, Khon Kaen University, Khon Kaen 40002, Thailand

**Keywords:** merlinoite, sugarcane bagasse ash, kaolin, lead, adsorption

## Abstract

This study introduces a merlinoite synthesized from sugarcane bagasse ash (SBA) and kaolin and evaluates its application as an adsorbent to remove lead from wastewater. The synthesis was performed via the hydrothermal method, and optimal conditions were determined. The adsorption of Pb by merlinoite was also optimized. Determination of the Pb^2+^ remaining in the aqueous solution was determined by atomic absorption spectroscopy (AAS). Adsorption isotherms were mainly studied using the Langmuir and Freundlich models. The Langmuir model showed the highest consistency for Pb adsorption on merlinoite, yielding a high correlation coefficient (R^2^) of 0.9997 and a maximum adsorption capacity (q_max_) of 322.58 mg/g. The kinetics of the adsorption process were best described by a pseudo-second-order model. Thermodynamic studies carried out at different temperatures established that the adsorption reaction was spontaneous and endothermic. The results of this study show that merlinoite synthesized from kaolinite and SBA is an excellent candidate for utilization as a high-performance adsorbent for lead removal from wastewater.

## 1. Introduction

Water pollution is a significant problem globally. This type of pollution is an increasing international concern, affecting economic growth and the health of people. Lead (Pb) is well known to be a highly toxic metal, and as such it is a pollutant of great concern. The release of Pb into the environment from industrial processes and the resulting impact on ecosystems and human health continues to be a serious issue [1]. The effects of lead toxicity are known to include kidney damage, brain damage, nervous system damage, anemia, and hypertension [2]. The World Health Organization (WHO) sets the maximum permissible level of Pb in drinking water at 0.01 mg/L [3]. The maximum allowable contaminant level of Pb in wastewater effluent from factories in Thailand is 0.2 mg/L, based on the Industrial Effluent Standard [4]. Strict and comprehensive management is essential for controlling the release of lead into the environment and preventing potentially devastating damage [1,2]. Lead can cause renal, gastrointestinal, and cardiovascular problems in humans. Various treatment techniques such as ion exchange [5], electrocoagulation [6], chemical precipitation [7], membrane separation [8], and adsorption [9] have been developed for removing lead from wastewater. However, adsorption is the most extensively used treatment technique because of its simplicity, effectiveness, and low cost [10]. The most common adsorbent materials are alumina, silica, activated carbon, chitosan, natural substances, and zeolites [10,11].

Zeolites are microporous crystalline alumina silicates in which SiO_4_ and AlO_4_ tetrahedra are covalently bound together by oxygen atoms [12]. The tetrahedra are arranged in three-dimensional frameworks. The net negative charge of the tetrahedral unit is balanced by exchangeable cations (K^+^, Na^+^, Ca^2+^) [12,13]. These cations can be exchanged with various heavy metals via the ion exchange reaction. Zeolites, formed in nature or synthesized from diverse sources, have a wide range of applications for purposes of adsorption, catalysis, and ion exchange [12,13]. Unfortunately, the synthesis of zeolites from commercial chemicals can be expensive [13]. Recently, there has been interest in using low-cost, readily available materials and waste materials from industry, such as natural clay [14], diatomite [15], coal fly ash [16], rice husk ash [17], and sugarcane bagasse fly ash [18]. Synthesizing zeolites from these types of materials has the added benefit of minimizing waste. In the current study, sugarcane bagasse ash (SBA) and kaolinite were selected as the raw materials for synthesizing zeolite, for environmental and economic reasons. SBA, mainly composed of SiO_2_, is a solid waste product produced extensively in Thailand, in amounts of approximately 651,000 tons per year, from the sugar industry and from electricity generation via bagasse–biomass fuel [19]. Kaolin, mainly composed of SiO_2_ and Al_2_O_3_, is a clay mineral that is both inexpensive and readily available [20].

Merlinoite is a small-pore zeolite containing interconnected eight-membered-ring pores. The crystallization system of this mineral is orthorhombic [21,22]. Synthetic merlinoite, also known as zeolite W, has a high capacity for ion exchange and adsorption. For example, the synthesis of merlinoite by conversion of coal fly ash with KOH solution has been reported [23]. The resultant merlinoite product demonstrated effectiveness as a fertilizer, and can be used for slow release of potassium into the soil. The preparation of merlinoite using the hydrothermal technique without an organic template was also investigated [24]. The merlinoite obtained in that study has a propensity for selecting K^+^ and is therefore useful for specifically extracting potassium from seawater. The fabrication of NH^4+^-loaded merlinoite by modifying K^+^-loaded merlinoite with ammonium ions was previously demonstrated [25]. This merlinoite is also suitable for extracting potassium from seawater. In addition, research based on the synthesis of two kinds of merlinoite with Si/Al ratios of 2.3 and 3.8 under completely inorganic conditions was undertaken. The prepared merlinoites demonstrated CO_2_ adsorption properties in their Na^+^, K^+^, Rb^+^, and Cs^+^ forms [26]. Thus, previous studies suggest that merlinoite could be a promising adsorbent for metal ion removal.

This study describes the synthesis of a merlinoite based on sugarcane bagasse ash (SBA) and kaolin with excellent adsorbent properties and the application of this environmentally friendly, low-cost adsorbent to Pb removal. Merlinoite can be synthesized easily via the hydrothermal route by combining the raw materials in a potassium hydroxide solution, which dissolves the various crystalline phases of the raw materials and then causes zeolite to precipitate out. The optimum conditions for hydrothermal synthesis of merlinoite from sugarcane bagasse ash and kaolin were studied. The lead adsorption capacity of merlinoite was investigated in terms of its adsorption isotherm, kinetics, and thermodynamics. Finally, the Pb adsorption capacity of merlinoite in wastewater was evaluated.

## 2. Materials and Methods

### 2.1. Materials

Sugarcane bagasse ash was provided by Kaset Thai Bio Power Co., Ltd., Nakhon Sawan, Thailand. Kaolin came from Ranong, Thailand. The chemical compositions of these materials, as determined by X-ray fluorescence spectrometry, are listed in Table S1. The following experiments also used potassium hydroxide (85%KOH, AR grade, Ajax Finechem, Seven Hills, NSW, Australia), lead (II) nitrate (99% Pb(NO_3_)_2_, AR grade, Merck, Darmstadt, Germany), Whatman filter paper (No. 5, Whatman, Kent, England), and deionized water.

### 2.2. Merlinoite Preparation

In this study, the merlinoite sample was prepared using the method that has been reported previously [23]. Sugarcane bagasse ash and kaolin were dried in an oven at 100 °C. The hydrothermal method was used for the synthesis of the merlinoite. Sugarcane bagasse ash, kaolin, and 2 M KOH (10 g:10 g:80 mL) were combined. The following parameters were investigated in order to determine optimal conditions for preparing the zeolite: reaction temperature (180 or 200 °C), reaction time (4, 8, 12, or 16 h), concentration of KOH solution (1, 2, 3, or 4 M), ratio of sugarcane bagasse ash to kaolin (1.1, 1:2, or 2:1), and ratio of raw materials to KOH solution (1:2, 1:3, 1:4, or 1:5). The mixture was placed in a stainless-steel PPL-lined autoclave and kept at the given temperature for the given amount of time in an oven under autogenous pressure. After synthesis, the material was washed with hot deionized water to remove excess KOH and then filtered. The material was dried at 105 °C for 16 h in an oven prior to analysis.

### 2.3. Characterization

The structural features of the merlinoite and the raw materials were analyzed using X-ray diffraction (XRD) (D2 PHASER, Bruker, Billerica, MA, USA). The X-ray diffraction patterns were recorded for a 2θ scan angle between 5 and 60°, with steps of 0.2° and a 0.02 deg/min scan rate. The chemical composition of the raw materials was analyzed using wavelength-dispersive X-ray fluorescence (WDXRF) (S8 TIGER, Bruker, Billerica, MA, USA). The surface topography and elemental analysis of merlinoite were investigated by scanning electron microscopy (SEM-EDS) (LEO 1455 VP, Leo, Sauerlach, Germany). Functional groups of merlinoite were identified by Fourier transform infrared spectroscopy (FTIR) (Spectrum GX, PerkinElmer, Waltham, MA, USA). Lead concentrations were determined by flame atomic absorption spectrophotometry (FAAS) (contrAA 800F, Analytik Jena, Jena, Thuringia, Germany).

### 2.4. Adsorption Experiments

The adsorption of Pb by the synthetic merlinoite zeolite was evaluated using a batch system. Different operating conditions were investigated including the solution pH (2–6), which was adjusted using 0.5 M HNO_3_ or 0.5 M NaOH, contact time (5–300 min), initial concentration (500–1200 ppm), and temperature (30–60 °C). In general, 50 mL of Pb solution was mixed with 0.1000 g of merlinoite using a temperature-controlled shaker (Thermo Fisher Scientific, Waltham, MA, USA) at a speed of 200 rpm. The suspension was then filtered using Whatman No. 5 filter paper. Flame atomic absorption spectrometry (FAAS) was used to determine the Pb concentration of the resulting supernatant. Air–acetylene was used as the flame type. A fuel flow of 65 L/h was used. A burner height of 5–9 mm was selected. The wavelength of 217 nm was used. All experiments were performed in triplicate, and the results were averaged.

The Pb adsorption percentage, Pb adsorption capacity (q_t_), and equilibrium amount of Pb adsorption (q_e_) were calculated using the following equations:

(1) $$\%\mathrm{Adsorption}=\;\frac{{(C}_{o}-C_{t})}{C_{o}}\;\times \;100$$

(2) $$q_{t}=\frac{{V(C}_{o}-C_{t})}{W}$$

(3) $$q_{e}=\frac{{V(C}_{o}-C_{e})}{W}$$

where C_o_ (mg/L) is the initial Pb concentration, C_t_ (mg/L) is the Pb concentration at time t, C_e_ (mg/L) is the equilibrium concentration, V (L) is the volume of the solution, and W (g) is the weight of merlinoite zeolite [27,28,29].

### 2.5. Application of Merlinoite for Pb Adsorption in Wastewater Samples

Wastewater samples were collected from three factories manufacturing batteries, paint, and religious statues in Samutprakarn and Phitsanulok, Thailand. The wastewater was filtered with Whatman No. 5 filter paper to eliminate suspended particles and then spiked with the stock Pb solution to a final Pb concentration of 50 ppm. A 50 mL sample and 0.1000 g of merlinoite were then mixed and shaken at 200 rpm on a temperature-controlled shaker at 30 °C for 150 min. After that, the suspension was filtered with Whatman No. 5 filter paper. FAAS was used to determine the Pb concentrations of both the initial solution and the supernatant that resulted after adsorption.

## 3. Results and Discussion

### 3.1. Merlinoite Synthesis Results

**Figure 1:**
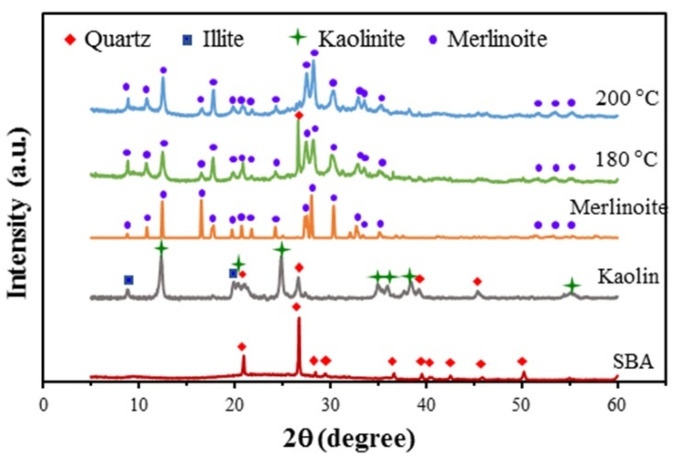
XRD patterns for the synthesized MZ at hydrothermal temperatures of 180 and 200 °C compared with the XRD patterns of a known merlinoite phase (JCPDS 29-0989) and the raw materials. Synthesis conditions: SBA/kaolin ratio = 1:1, raw material/2 M KOH ratio = 1:4.

The synthesis of merlinoite from SBA and kaolin via hydrothermal conversion was found to be affected by the synthesis conditions investigated in this study: the hydrothermal temperature, hydrothermal time, concentration of KOH solution, ratio of sugarcane bagasse ash to kaolin, and ratio of raw materials to KOH solution. Figure 1 shows the XRD patterns for the merlinoite synthesized at hydrothermal temperatures of 180 and 200 °C, compared with the XRD patterns of the reference merlinoite phase (JCPDS 29-0989) and the two raw materials. All diffraction peaks of the MZ synthesized at 200 °C corresponded perfectly to those of the reference merlinoite phase, indicating complete conversion to merlinoite from the raw materials. However, for the merlinoite synthesized at 180 °C, there was a peak remaining from the raw materials, corresponding to the quartz phase at 26.7°, indicating incomplete conversion at the lower temperature. Thus 200 °C was used as the temperature for subsequent hydrothermal synthesis.

**Figure 2:**
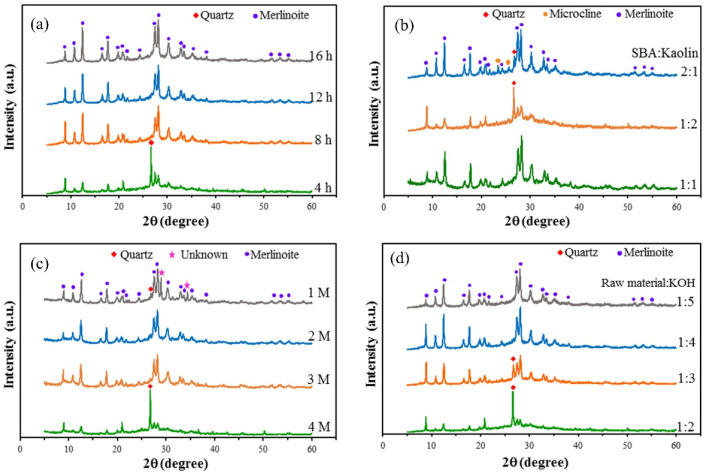
XRD patterns for the synthesized MZ formed at: (**a**) reaction times of 4, 8, 12, and 16 h (synthesis conditions: SBA/kaolin ratio = 1:1, raw material/2M KOH ratio = 1:4, hydrothermal temperature = 200 °C); (**b**) SBA/kaolin ratios of 1:1, 1:2, and 2:1 (synthesis conditions: raw material/2M KOH ratio = 1:4, hydrothermal temperature = 200 °C, reaction time = 8 h); (**c**) KOH concentrations of 1, 2, 3, and 4 M (synthesis conditions: SBA/kaolin ratio = 1:1, raw material/KOH solution ratio = 1:4, hydrothermal temperature = 200 °C, reaction time = 8 h); (**d**) raw material to KOH ratios of 1:2, 1:3, 1:4, and 1:5 (synthesis conditions: SBA/kaolin ratio = 1:1, raw material/2M KOH ratio = 1:4, hydrothermal temperature = 200 °C, reaction time = 8 h).

Figure 2a shows the XRD patterns for the merlinoite prepared using reaction times of 4, 8, 12, and 16 h. Unconverted quartz was still detected at 4 h, but single-phase merlinoite formed at 8, 12, and 16 h. The reaction time of 8 h was selected as optimal, since this was sufficient time for the reaction to be completed. Figure 2b shows the XRD patterns for the merlinoite synthesized using SBA/kaolin ratios of 1:1, 1:2, and 2:1. The merlinoite formed with high intensity and phase purity when an SBA/kaolin ratio of 1:1 was used but using a ratio of 1:2 resulted in low intensity and the presence of a quartz phase as an impurity. Merlinoite formed with high intensity when an SBA/kaolin ratio of 2:1 was used; however, there were also quartz and microcline phases. An SBA/kaolin ratio of 1:1 was thus chosen as optimal for the synthesis of highly crystalline merlinoite. Figure 2c shows the XRD patterns for the merlinoite synthesized using KOH concentrations of 1, 2, 3, and 4 M. The results show that raising the KOH concentration to 2 M caused complete dissolution of quartz, and only merlinoite was detected. Furthermore, at 4 M, an additional unknown phase emerged. The principle of minimal chemical use makes 2 M the optimal KOH concentration for merlinoite synthesis. Figure 2d shows the XRD patterns for the merlinoite synthesized using raw-material-to-KOH ratios of 1:2, 1:3, 1:4, and 1:5. A quartz phase was still detected at ratios of 1:2 and 1:3. Single-phase merlinoite was obtained at ratios of 1:4 and 1:5. Choosing the minimal amount of chemicals, a ratio of 1:4 was used for merlinoite synthesis.

### 3.2. Characterization of Merlinoite

**Figure 3:**
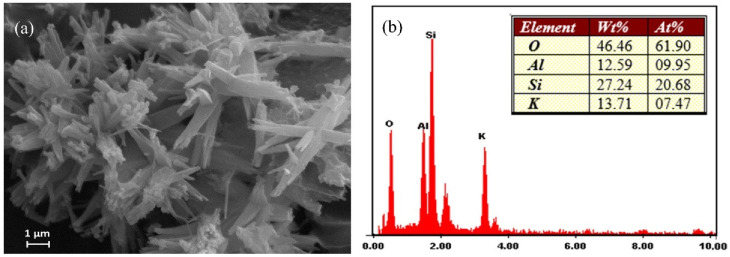
(**a**) SEM image of the synthesized merlinoite at 5000× magnification and (**b**) EDX analysis of the elemental composition.

The morphology of the synthetic merlinoite was examined by SEM. Figure 3 shows the SEM photos at 5000× magnification. The morphology was characterized by bundles of columns with uneven lengths. EDS was used to analyze the synthetic merlinoite’s elemental components. The constituent elements are shown by weight percent in Figure 3b. The results indicate that the merlinoite consists mainly of silicon, aluminum, potassium, and oxygen.

In addition, it should be noted that the Al/Si ratio was quite small (0.46). However, even using this low Al/Si ratio, the resultant merlinoite still showed a high Pb removal efficiency, as shown later. This agrees well with results reported in the literature [25,30].

The TEM micrographs (Figure S1a,b) of the synthesized merlinoite showed column-like morphology. The SEAD pattern in Figure S1c exhibits a ring-like pattern suggesting the monocrystalline nature of the sample [31]. In addition, the HR-TEM micrograph in Figure S1d shows the lattice fringe with a d-spacing of 0.54 nm. This is due to the reflection from the (121) plane of the merlinoite.

The specific surface area is an important factor affecting the adsorption efficiency of an adsorbent [32]. Therefore, the textural properties of the synthesized merlinoite were investigated. The nitrogen adsorption–desorption isotherms and the BJH pore size distribution of the sample (Figure S2) showed a type IV isotherm following the IUPAC classification. A BET surface area of about 20 m^2^/g was obtained. The sample showed the mesoporous nature of the resultant merlinoite.

The FTIR spectrum of the synthesized merlinoite is shown in Figure S3. The bands in the range of 3428–3608 cm^−1^ are valence vibrations of O-H bonds. The O-H band at 1635 cm^−1^ was assigned to deformation vibrations of adsorbed water molecules. The strongest peak at around 1007 cm^−1^ was seen in zeolite as asymmetric stretching vibrations of Si–O–Si and Si–O–Al, whereas the peak at about 753 cm^−1^ represented the symmetric stretching vibration. The peak at 427 cm^−1^ was attributed to the internal vibration of Si–O–Si and Si–O–Al bending [33]. These spectra indicate that the synthesized product had Si–O–Si or Si–O–Al groups, as a specific fingerprint of zeolite. In addition, it should be noted that the FTIR spectra of the fresh and the used merlinoite (after Pb adsorption) are similar. However, the slight lowering of the transmittance after Pb adsorption correlates well with that shown in previous work [27], where a small vibrational band shift was also detected, indicating the attachment of Pb^2+^ on the adsorbent.

### 3.3. Adsorption Behavior

#### 3.3.1. Effect of Solution pH

The pH is an important factor affecting adsorption capacity because the solution pH influences both the surface properties of the merlinoite and the ionic forms of the lead ions in solution. The effect of pH on Pb adsorption is displayed in Figure S4. The results show that Pb adsorption was poor at low pH levels. At low pH, the merlinoite surface functional groups are protonated, leading to many positively charged species and a decrease in the number of active adsorption sites [34]. Electrostatic repulsion between the positively charged functional groups and Pb could also inhibit the binding of Pb onto the surface of the merlinoite, thus lowering adsorption. When the pH is higher, there are more deprotonated functional groups and thus more active adsorption sites available. This promotes enhanced interaction between Pb and the functional groups, so higher levels of Pb removal can be expected [35]. The optimal initial solution pH for Pb adsorption was found to be pH 6, which yielded 93.63% Pb removal. On the other hand, using a pH higher than 6 causes Pb precipitation [28,36].

#### 3.3.2. Effect of Contact Time

The effect of contact time on Pb adsorption is shown in Figure S5. The results show that as Pb contact time increased from 5 to 150 min, adsorption also increased from 81% to 99%, leveling off after that. Initially, vacant active sites are abundantly available on the merlinoite surface, so the uptake of Pb can occur readily. At around 150 min, the situation changes. The vacant active sites have been filled, and equilibrium has been reached. Thus, a contact time of 150 min was selected for use in the subsequent experiments.

#### 3.3.3. Effect of Co-Existing Metal Ions

The effect of various co-existing metal ions on Pb adsorption is shown in Figure S6. The results show that the adsorbent is selective for removing Pb^2+^ in the solution, in comparison to other metal ions. The effect of the co-existing metal ions on lead removal can therefore be neglected. An efficiency of about 80–98% was achieved.

#### 3.3.4. Adsorption Kinetics

The adsorption kinetics and rate constants of Pb adsorbed on merlinoite were determined using kinetic models. In investigations based on equilibrium adsorption, the pseudo-first-order and pseudo-second-order kinetic models are expressed as follows:

ln(q_e_ − q_t_) = ln q_e_ − k_1_t (4)

(5) $$\frac{1}{q_{t}}=\frac{t}{q_{e}}+\frac{1}{k_{2}q_{e}^{2}}$$

where q_e_ and q_t_ represent the amounts of Pb ions adsorbed onto the merlinoite (mg/g) at equilibrium and at time t, respectively, and k_1_ and k_2_ are the rate constants for the pseudo-first-order and pseudo-second-order kinetics, respectively [37].

The linear plots of the pseudo-first-order and pseudo-second-order models for Pb adsorption process are shown in Figure S7, and the model parameters obtained from the fitting are listed in Table 1. It was observed that the coefficient of regression value (R^2^) determined from the pseudo-second-order model (0.9999) was higher than that from the pseudo-first-order model (0.8430). In addition, the experimental adsorption capacity (q_e, exp_) of 247.21 mg/g clearly fitted well with the calculated adsorption capacity (q_e,cal_) of 250.00 mg/g from the pseudo-second-order model. The results indicate that Pb adsorption onto merlinoite can be described very well using a pseudo-second-order model, which means the kinetics of adsorption are those of chemical adsorption. The positively charged Pb cation reacts with negatively charged terminal hydroxyl groups on the merlinoite surface.

#### 3.3.5. Adsorption Isotherms

The adsorption isotherm shows the distribution of the adsorbate molecules at the liquid/adsorbent interface. In this study, the Langmuir and Freundlich isotherm models were investigated to characterize the Pb adsorption process on the merlinoite. The experimental data were fitted using these isotherms (Figure S8), and the obtained parameters are presented in Table 2. The linear forms of the Langmuir and Freundlich isotherms are shown below in Equations (6) and (7).

(6) $$\frac{C_{e}}{q_{e}}=\frac{C_{e}}{q_{m}}+\frac{1}{{(q}_{m}K_{L})}$$

(7) $${\mathrm{lnq}}_{e}{=\mathrm{lnK}}_{F}+\frac{1}{n}{\;(\mathrm{lnC}}_{e})$$

where C_e_ is the concentration of Pb solution at equilibrium (mg/L), q_e_ is the quantity of Pb adsorbed by the merlinoite (mg/g), q_m_ is the maximum adsorption capacity (mg/g), and K_L_ is the Langmuir constant (L/mg). The terms K_F_ and n are Freundlich constants which represent the adsorption capacity and the intensity of adsorption, respectively [38].

The experimental data clearly agree with the Langmuir adsorption isotherm (R^2^ = 0.9997) better than with the Freundlich adsorption isotherm (R^2^ = 0.9248), so adsorption of Pb should take place in a monolayer on the surface of merlinoite. In addition, the favorability of the adsorption process can be predicted by the separation factor (R_L_), which can be calculated using Equation (8). The R_L_ values for all Pb concentrations were found to be in the range of 0.002–0.005, indicating the favorability of the Langmuir isotherm.

(8) $$R_{L}=\frac{1}{{(1+K}_{L}C_{o})}$$

where K_L_ is the Langmuir constant and C_o_ (mg/L) is the initial Pb concentration. The value of R_L_ predicts the adsorption process: unfavorable (R_L_ > 1), favorable (0 < R_L_ < 1), linear (R_L_ = 1) or irreversible (R_L_ = 0) [39].

For comparison, Table S2 shows the q_m_ values for various adsorbents used for Pb adsorption in previous studies. The merlinoite investigated in this study has superior adsorption capacity.

Furthermore, apart from these two models, other adsorption models were also used to fit the raw data regarding Pb adsorption [40,41,42,43]. These models included the Redlich–Peterson isotherm, the Temkin isotherm, and the Dubinin–Radushkevich isotherm. The results are shown in Figure S8. However, the Langmuir model still showed the best fit.

#### 3.3.6. Adsorption Thermodynamics

Determination of the thermodynamic parameters is vital to understanding the spontaneity and feasibility of an adsorption process. These parameters include the change in Gibb’s free energy of adsorption (∆G°), the change in enthalpy of adsorption (∆H°), and the change in entropy of adsorption (∆S°), which were determined using Equations (9)–(12), respectively [34,35,44,45].

K_e_ = q_e_/C_e_ (9)

∆G° = −RTlnK_e_ (10)

∆G° = ∆H° − T∆S°
(11)

(12) $${\mathrm{lnK}}_{e}=\frac{\Delta S^{o}}{R}\frac{\Delta H^{o}}{\mathrm{RT}}$$

where K_e_ represents the equilibrium distribution constant, R is the ideal gas constant (8.314 J/K ·mol), and T(K) is the temperature. The adsorption thermodynamics model was established by plotting the value of ln(q_e_/C_e_) against the reciprocal of temperature (1/T), to calculate the values of ∆H° and ∆S° as well as the regression coefficient (R^2^), as shown in Figure S9.

The adsorption thermodynamic parameters are summarized in Table 3. The positive value of ∆H° (40.55 kJ/mol) indicates an endothermic process of Pb adsorption onto merlinoite. The negative value of ∆G° over all the studied temperatures indicates a spontaneous process, and the degree of spontaneity of the adsorption process increases with increasing temperature. As the temperature increases, the mobility of Pb increases and the retardation of the diffusing ions decreases. The positive value of ∆S indicates that an increase in the randomness of the adsorbed Pb at the merlinoite–solution interface during the adsorption is expected.

#### 3.4. Application of Merlinoite for Lead Adsorption in Wastewater Samples

Table 4 shows the results of utilizing the merlinoite product in actual wastewater samples with 50 ppm Pb. The adsorption percentages of merlinoite in all the wastewater samples were in the range of 94.4–97.6%. The results indicate that the synthesized merlinoite is an effective adsorbent for the removal of Pb from wastewater. In addition, comparison of the lead adsorption found in the present work and those reported in the literatures [46,47,48,49,50,51] can be shown in Table S2.

### 4. Conclusions

Merlinoite was successfully synthesized from sugarcane bagasse ash and kaolin using a one-step hydrothermal method. The adsorption of lead by the merlinoite depended on the solution pH, contact time, lead concentration, and temperature. The experimental data in the adsorption process correlated well with the pseudo-second-order kinetic model and the Langmuir adsorption isotherm. The maximum monolayer adsorption capacity of 322.58 mg/g obtained from merlinoite was higher than that obtained from other adsorbents. The thermodynamic data revealed that the lead adsorption was a spontaneous endothermic process. Thus, this new merlinoite, synthesized inexpensively from kaolinite and sugarcane bagasse ash, is an excellent candidate for utilization as a high-performance adsorbent in the removal of lead from wastewater.

## Figures and Tables

**Table 1:** Adsorption kinetics parameters of Pb adsorption onto merlinoite.

q_e, exp_ (mg/g)	Pseudo-First-Order Model			Pseudo-Second-Order Model		
	k_1_ (1/min)	q_e,cal_ (mg/g)	R^2^	k_2_ (g/mg min)	q_e,cal_ (mg/g)	R^2^
247.21	0.0144	27.17	0.8430	0.0016	250.00	0.9999

**Table 2:** Isotherm parameters for the adsorption of Pb on merlinoite.

Langmuir Isotherm			Freundlich Isotherm		
**q_m_ (mg/g)**	**K_L_ (L/mg)**	**R^2^**	**n**	**K_F_ (L/mg)**	**R^2^**
322.58	0.344	0.9997	18.01	231.15	0.9248

**Table 3:** Thermodynamic parameters for adsorption of Pb on merlinoite.

∆G° (kJ/mol)			∆H° (kJ/mol)	∆S° (J/mol·K)
303 K	313 K	333 K		
−5.938	−7.557	−10.562	40.55	152.67

**Table 4:** Pb adsorption capacity of synthesized merlinoite in various wastewater samples.

Wastewater Sample Type	Initial Concentration (mg/L)	Equilibrium Concentration (mg/L)	Adsorption (%)
Battery factory +50 ppm Pb	50.0	2.1	95.8
Painting factory +50 ppm Pb	50.0	1.2	97.6
Religious statues factory +50 ppm Pb	50.0	2.8	94.4

## Data Availability

Not applicable.

## Supplementary Materials

References [46,47,48,49,50,51] are cited in the Supplementary Materials. The characterization of the samples and also the lead removal capacities can be found in the supporting information. The following are available online, Figure S1: TEM micrographs (**a** and **b**), SAED pattern (**c**) and HR-TEM micrograph of the merlinoite. Figure S2: N2 adsorption–desorption isotherms (**a**), BJH pore size distributions (**b**) of merlinoite. Figure S3: FT-IR spectra of the synthetic merlinoite (before and after Pb adsorption). Figure S4: Effect of pH on Pb adsorption by merlinoite (Pb concentration = 500 mg/L, merlinoite wt. = 0.1000 g, time = 150 min, temperature = 30 °C). Figure S5: Effect of contact time on Pb adsorption by merlinoite (Pb concentration = 500 mg/L, merlinoite weight. = 0.1000 g, pH = 6, temperature = 30 °C). Figure S6: Effect of co-existing metal ions on Pb adsorption by merlinoite (Pb concentration = 500 mg/L, merlinoite weight. = 0.1000 g, pH = 6, temperature = 30 °C). Figure S7: Kinetic model graphs: (**a**) pseudo-first-order model and (**b**) pseudo-second-order model for Pb adsorption onto merlinoite. (Pb concentration = 500 mg/L, merlinoite = 0.1000 g, pH = 6, temperature = 30 °C). Figure S8: Plots of (**a**) the Langmuir isotherm, (**b**) the Freundlich isotherm, (**c**) the Redlich-Peterson isotherm, (**d**) Temkin isotherm and (**e**) Dubinin-Radushkevich isotherm for Pb adsorption onto merlinoite. (initial Pb concentration = 500–1200 mg/L, merlinoite weight = 0.1000 g, pH = 6, time = 150 min, temperature = 30 °C). Figure S9: Graph of adsorption thermodynamics for Pb adsorption onto merlinoite. (Pb concentration = 600 ppm, merlinoite weight = 0.1000 g, pH = 6, time = 150 min). Table S1: Chemical composition of sugarcane bagasse ash and kaolin (%wt). Table S2: Comparing adsorption capacities of various adsorbents for Pb.
