# Supplementary material for: Removal of Lead by Merlinoite Prepared from Sugarcane Bagasse Ash and Kaolin: Synthesis, Isotherm, Kinetic, and Thermodynamic Studies
Source: Molecules. 2021 Dec 13;26(24):7550. doi: 10.3390/molecules26247550 (PMC8709325; doi:10.3390/molecules26247550)
Supplement: Supplementary file 1 [file molecules-26-07550-s001.zip › molecules-1365650-supplementary.pdf]

Supplementary Information

Removal of lead by merlinoite prepared from sugarcane bagasse ash and kaolin: synthesis, isotherm, kinetic and thermodynamic studies

Tussaneetorn Chuenpratoom[1], Khuanjit Hemavibool[1], Kritsana Rermthong[1], Suwat Nanan,[2*]

1 Department of Chemistry, Faculty of Science, Naresuan University, Phitsanulok 65000, Thailand.

2 Materials Chemistry Research Center, Department of Chemistry and Center of Excellence for Innovation in Chemistry (PERCH-CIC), Faculty of Science, Khon Kaen University, Khon Kaen, 40002, Thailand.

*Corresponding Author: suwatna@kku.ac.th

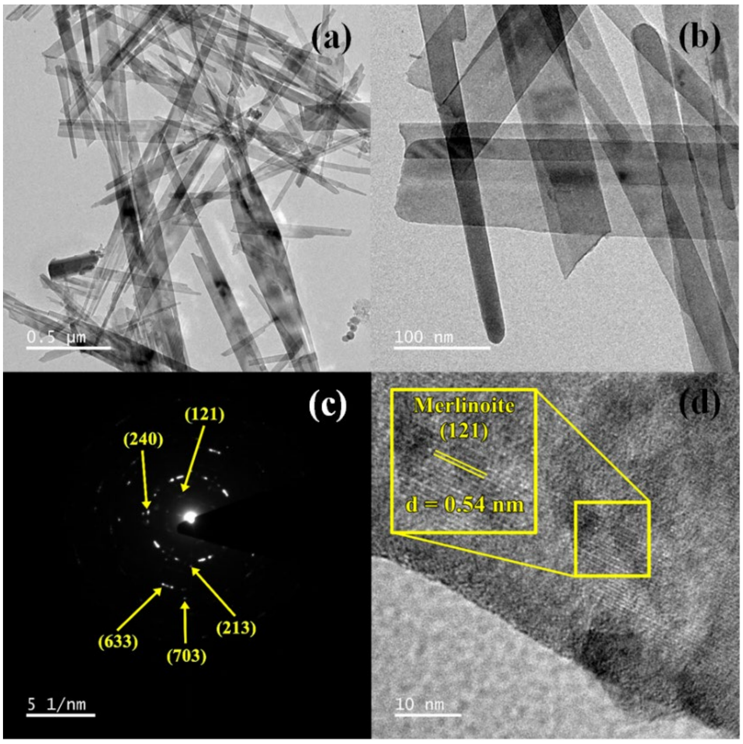

Figure S1 TEM micrographs (a and b), SAED pattern (c) and HR-TEM micrograph of the merlinoite.

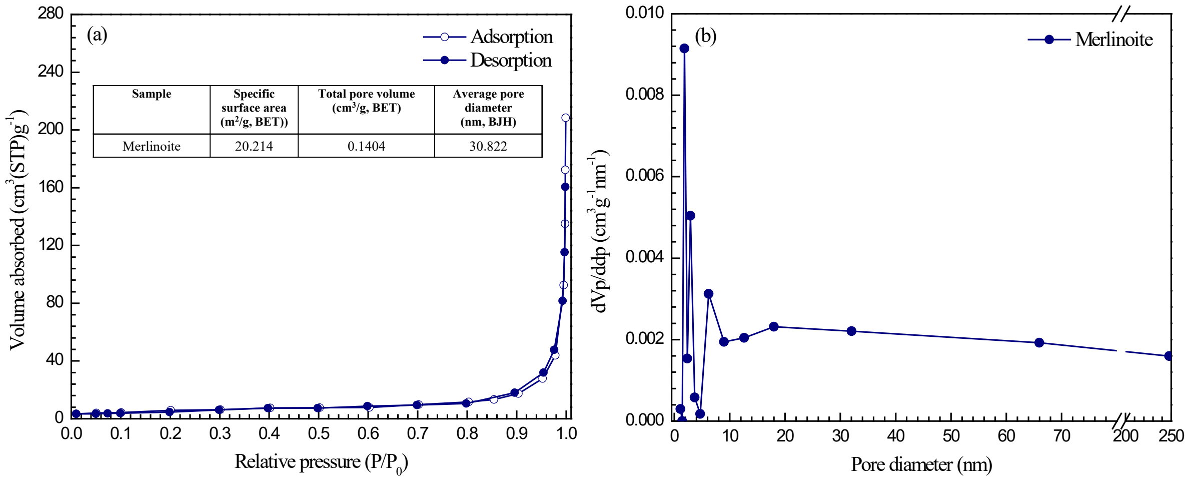

Figure S2 N_2 adsorption–desorption isotherms (a), BJH pore size distributions (b) of merlinoite.

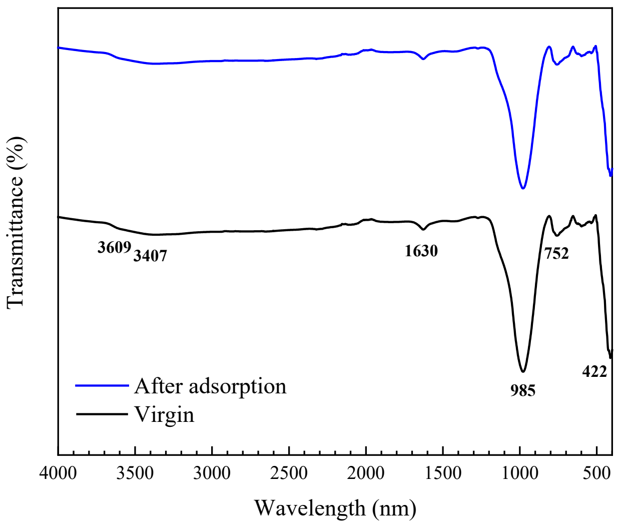

Figure S3 FT-IR spectra of the synthetic merlinoite (before and after Pb adsorption).

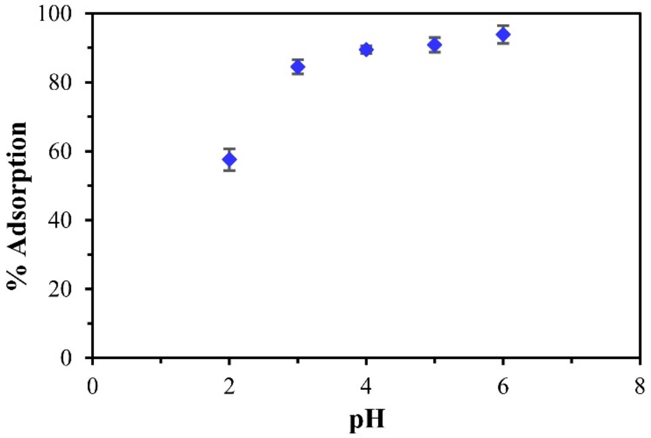

Figure S4 Effect of pH on Pb adsorption by merlinoite (Pb concentration = 500 mg/L, merlinoite wt. = 0.1000 g, time = 150 min, temperature = 30 °C).

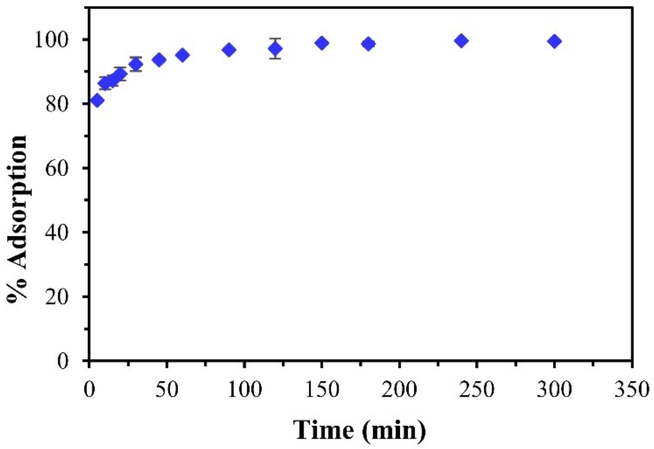

Figure S5 Effect of contact time on Pb adsorption by merlinoite (Pb concentration = 500 mg/L, merlinoite weight. = 0.1000 g, pH = 6, temperature = 30 °C).

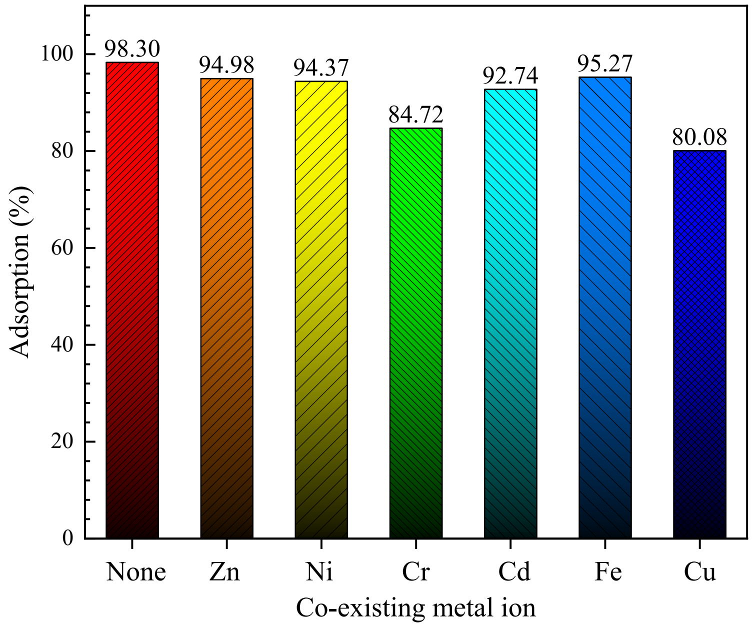

Figure S6 Effect of co-existing metal ions on Pb adsorption by merlinoite (Pb concentration = 500 mg/L, merlinoite weight. = 0.1000 g, pH = 6, temperature = 30 °C).

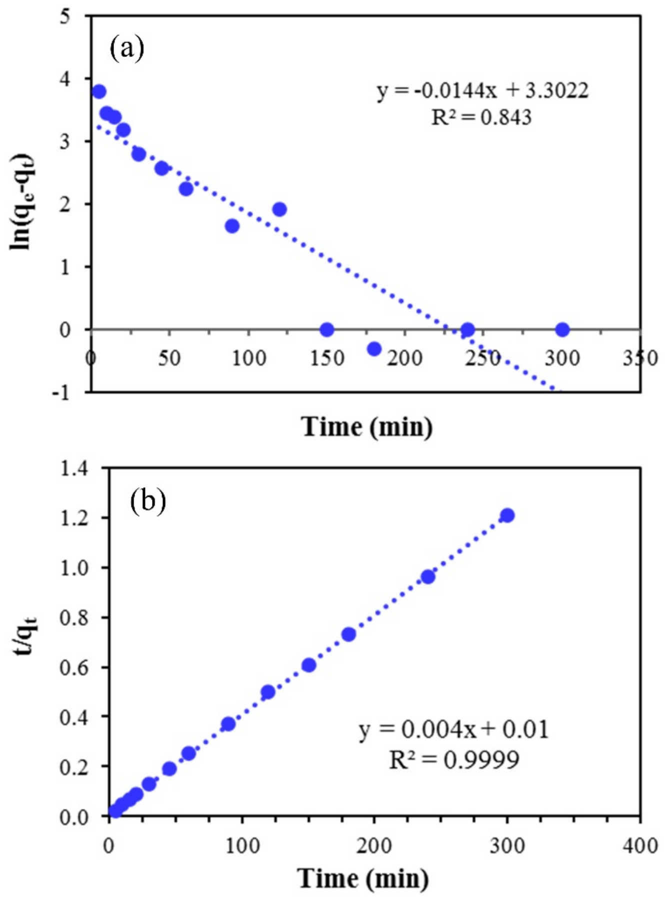

Figure S7 Kinetic model graphs: (a) pseudo-first-order model and (b) pseudo-second-order model for Pb adsorption onto merlinoite. (Pb concentration = 500 mg/L, merlinoite = 0.1000 g, pH = 6, temperature = 30 °C).

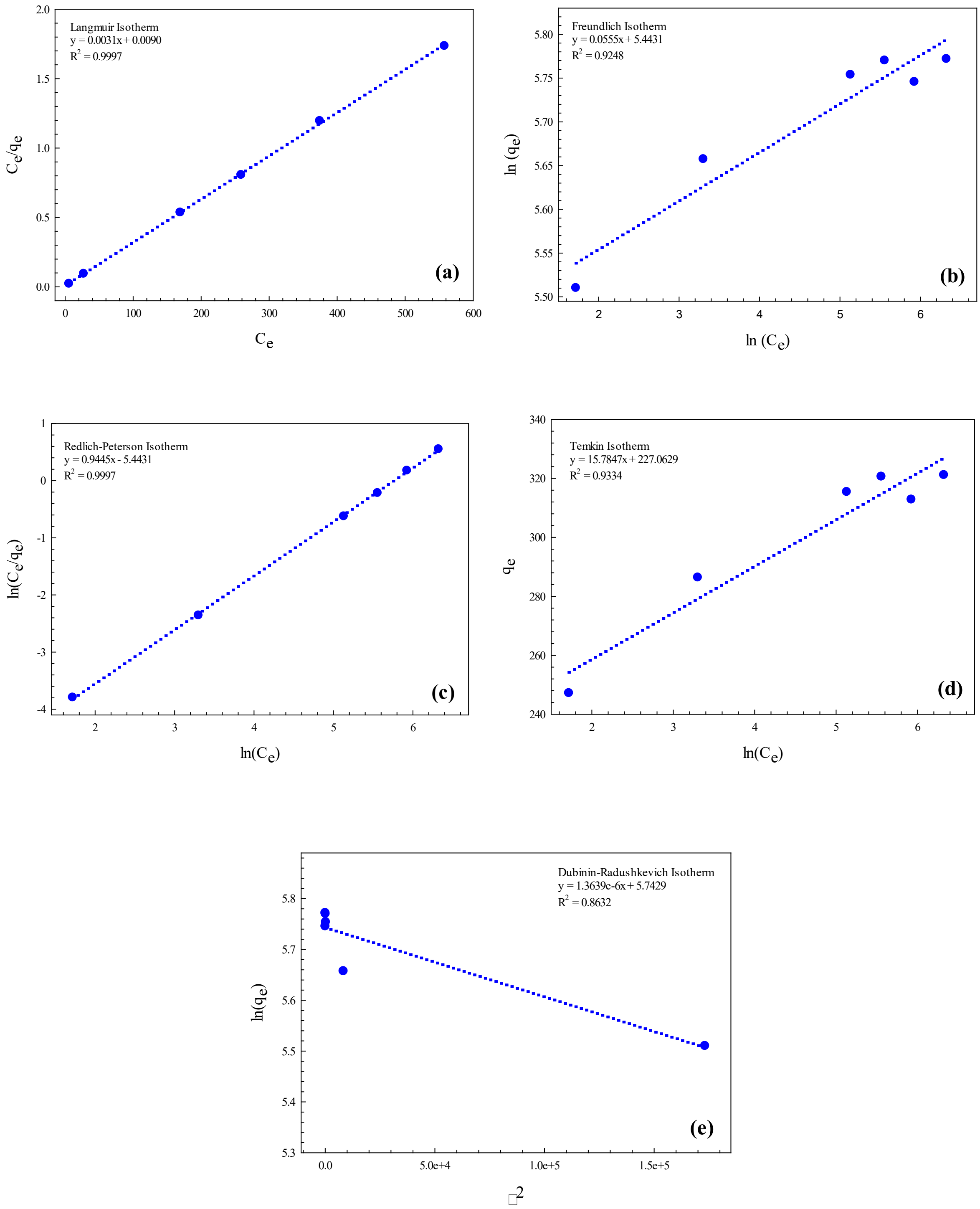

Figure S8 Plots of (a) the Langmuir isotherm, (b) the Freundlich isotherm, (c) the Redlich-Peterson isotherm, (d) Temkin isotherm and (e) Dubinin-Radushkevich isotherm for Pb adsorption onto merlinoite. (initial Pb concentration = 500-1200 mg/L, merlinoite weight = 0.1000 g, pH = 6, time = 150 min, temperature =30 °C).

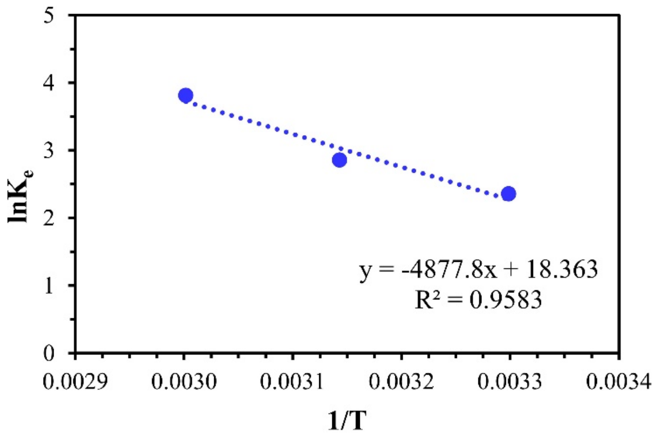

Figure S9 Graph of adsorption thermodynamics for Pb adsorption onto merlinoite. (Pb concentration = 600 ppm, merlinoite weight = 0.1000 g, pH = 6, time = 150 min).

Table S1. Chemical composition of sugarcane bagasse ash and kaolin (%wt).

Material	SiO_2	Al_2O_3	Fe_2O_3	CaO	K_2O	MgO	Na_2O	TiO_2	SO_3	P_2O_5	MnO
bagasse ash	63.60	6.47	3.51	5.90	2.94	1.70	0.022	0.50	0.87	0.90	0.24
kaolin	47.90	36.60	0.80	0.25	1.94	0.51	1.94	0.07	-	-	-

Table S2. Comparing adsorption capacities of various adsorbents for Pb.

Sample type	Adsorbent	Adsorption capacity (mg/g)	Reference
Zeolite	Merlinoite	322.58	This study
Nanocomposite	Zeolite/Zinc Oxide	47.6	[1]
Bionanocomposite	Xanthan Gum-Glutathione/Zeolite	109.01	[2]
Zeolite	Potassium ore leaching residue	25.88	[3]
Zeolite	NaP1 and faujasite	144.7191	[4]
Zeolite	K-type	102.0	[5]
Zeolite	Modified Natural Zeolite	258.71	[6]

References

[1] Alswata, A.A., Ahmad, M.B., Al-Hada, N.M., Kamari, H.M., Hussein, M.Z.B., Ibrahim, N.A., 2017. Preparation of Zeolite/Zinc Oxide Nanocomposites for toxic metals removal from water. Results Phys. 7, 723-731. https://doi.org/10.1016/j.rinp.2017.01.036.

[2] Ahmad, R., Mirza, A., 2018. Adsorptive removal of heavy metals and anionic dye from aqueous solution using novel Xanthan gum-Glutathione/ Zeolite bionanocomposite. Groundw. Sustain. Dev. 7, 305-312. https://doi.org/10.1016/j.gsd.2018.07.002.

[3] Xing, P., Wang, C.Y., Ma, B.Z., Chen, Y.Q., 2018. Removal of Pb(II) from aqueous solution using a new zeolite-type absorbent: Potassium ore leaching residue. J. Environ. Chem. Eng. 6(6), 7138-7143. https://doi.org/10.1016/j.jece.2018.11.003.

[4] Utami, A.R., Sugiarti, S., Sugita, P., 2019. Synthesis of NaP1 and faujasite zeolite from natural zeolite of Ende-NTT as lead (Pb(II))adsorbent. Rasayan J. Chem. 12(2), 650-658. https://doi.org/10.31788/RJC.2019.1222056.

[5] Kobayashi, Y., Ogata, F., Saenjum, C., Nakamura, T., Kawasaki, N., 2020. Removal of Pb^{2+} from Aqueous Solutions Using K-Type Zeolite Synthesized from Coal Fly Ash. Water. 12(9), 2375. https://doi.org/10.3390/w12092375.

[6] Rahimi, M., Mahmoudi, J., 2020. Heavy Metals Removal from Aqueous Solution by Modified Natural Zeolites Using Central Composite Design. Period. Polytech. Chem. Eng. 64(1), 106-115. https://doi.org/10.3311/PPch.13093.
